# Spatially Adjusted Time-varying Reproductive Numbers: Understanding the Geographical Expansion of Urban Dengue Outbreaks

**DOI:** 10.1038/s41598-019-55574-0

**Published:** 2019-12-16

**Authors:** Ta-Chou Ng, Tzai-Hung Wen

**Affiliations:** 1 Graduate Institute of Epidemiology and Preventive Medicine, College of Public Health, National Taiwan University, Taipei, 100 Taiwan; 2 Department of Geography, National Taiwan University, Taipei City, 106 Taiwan

**Keywords:** Infectious diseases, Epidemiology, Computational science

## Abstract

The basic reproductive number (*R*_0_) is a fundamental measure used to quantify the transmission potential of an epidemic in public health practice. However, *R*_0_ cannot reflect the time-varying nature of an epidemic. A time-varying effective reproductive number *R*_*t*_ can provide more information because it tracks the subsequent evolution of transmission. However, since it neglects individual-level geographical variations in exposure risk, *R*_*t*_ may smooth out interpersonal heterogeneous transmission potential, obscure high-risk spreaders, and hence hamper the effectiveness of control measures in spatial dimension. Therefore, this study proposes a new method for quantifying spatially adjusted (time-varying) reproductive numbers that reflects spatial heterogeneity in transmission potential among individuals. This new method estimates individual-level effective reproductive numbers (*R*^*j*^) and a summarized indicator for population-level time-varying reproductive number (*R*_*t*_). Data from the five most severe dengue outbreaks in southern Taiwan from 1998–2015 were used to demonstrate the ability of the method to highlight early spreaders contributing to the geographic expansion of dengue transmission. Our results show spatial heterogeneity in the transmission potential of dengue among individuals and identify the spreaders with the highest *R*^*j*^ during the epidemic period. The results also reveal that super-spreaders are usually early spreaders that locate at the edges of the epidemic foci, which means that these cases could be the drivers of the expansion of the outbreak. Therefore, our proposed method depicts a more detailed spatial-temporal dengue transmission process and identifies the significant role of the edges of the epidemic foci, which could be weak spots in disease control and prevention.

## Introduction

The basic reproductive number (*R*_0_) is a fundamental measure used to quantify the transmission potential of an epidemic^1^. It is defined as the number of infections caused by an index case within a completely susceptible population, i.e., a population in which there is no pre-existing immunity. *R*_0_ is a summary index suggesting both the intrinsic transmissibility of a pathogen and the infrastructure that allows the disease to spread in a given setting. In particular, the value of *R*_0_ is affected by transmission probability, contact rate and duration of infectiousness^2^.

Based on deterministic homogenous-mixing epidemic models^3^, public health practitioners usually regard R_0_ = 1 as a useful threshold for ensuring the development of an outbreak, referred to as the epidemic threshold^4^. The health authorities also use *R*_0_ for predicting final epidemic size^3,5^ and assessing the resources required to contain the outbreak, e.g., determining what proportion of people should be vaccinated^2^. Therefore, it is of great practical importance to estimate *R*_0_ for a possible outbreak in public health practice. The value of *R*_0_ is estimated from the initial growth rate of an epidemic based on mechanistic models, such as the susceptible-infected-recovered model^3,6^. For example, *R*_0_ has been estimated at approximately 1.5 for 2009 H1N1 influenza^7^, at 3 for the 2003 severe acute respiratory syndrome (SARS) outbreak^8^, and at 12–18 for historical measles outbreaks^9^. As an epidemic unfolds, however, the depletion and recovery of the susceptible population cause fluctuating effective transmissibility, so the value of *R*_0_ cannot reflect the time-varying nature of an epidemic. Moreover, when an epidemic occurs in a realistic contact network, *R*_0_ can also fail to characterize the transmission potential at the initial stage^10^.

On the other hand, the effective reproductive number (*R*) is defined as the number of infections caused by any case^11^. The difference between *R* and *R*_0_ is that the value of *R* does not depend on the assumption that the population is completely susceptible, which is often violated in later stages of an outbreak or in a situation in which the population has been exposed to the pathogen previously. Therefore, *R* aims to characterize the progression of an epidemic in a realistic scenario. Intuitively, counting the branches of infection on transmission trees (i.e., who infects whom^6^) precisely quantifies the value of *R*; however, doing this is impractical in most circumstances except for confined outbreaks where contact tracing is feasible^12^. The epidemic curve (an illustration of the number of new infections) does not encode transmission dynamics and cannot be used to infer *R* directly. The *renewal equation*^11^, which describes the temporal transmission relationship between propagating generations of infected cases, is therefore proposed to estimate *R* from incidence data in different epidemic periods. More specifically, the renewal equation estimates the values of the time-varying effective reproductive number (*R*_*t*_), defined as the population-level transmission potential at time *t*^11,13^. In a fully susceptible population, early values of *R*_*t*_ should approximate *R*_0_, but *R*_*t*_ is more informative in that it tracks the subsequent evolution of transmission potential during the course of an outbreak. The values of *R*_*t*_ can also reveal the time when an outbreak was contained by monitoring the epidemic threshold. During the 2014 Ebola outbreak in West Africa, *R*_*t*_ captured a distinct temporal pattern of transmission potential in different countries, signaling the different levels of control measures needed^14^. As the change in transmission potential is highly correlated to control measures, public health practitioners can evaluate the effectiveness of control measures by determining the change in *R*_*t*_ after implementation. For example, sudden drops in *R*_*t*_ (from 3 to approximately 0.7) after the issuance of a global alert during the 2003 SARS outbreak indicated the effectiveness of the alert^15^.

**Figure 1:**
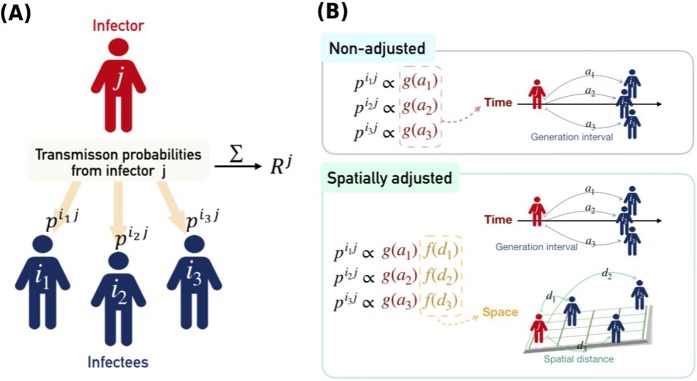
Illustration of how individual reproductive numbers were calculated (panel A) and the difference between the spatially adjusted and non-adjusted methods (panel B). The transmission probability from individual *j* to all potential infectees $({p}^{{i}_{1}j},{p}^{{i}_{2}j},{p}^{{i}_{3}j})$ is estimated first. The sum of these probabilities is, by definition, the expected number of infectees caused by individual *j*, i.e., the individual reproductive number. The transmission probability itself is estimated based on solely temporal relationships (non-adjusted) or in combination with spatial relationships (spatially adjusted). In the non-adjusted method, *p*^·*j*^ is proportional to the temporal weight *g*(*a*) determined by the generation interval between infector *j* and its infectees. Cases in the same temporal cohort (i.e., with the same onset day, *a*_1_ = *a*_2_ = *a*_3_) share the same transmission probability from previous infectors. They also share the same individual reproductive number since their relationship to subsequent cases is again identical. For the spatially adjusted method, *p*^·*j*^ is proportional to the spatial weights *f*(*d*), modulated by the distance between the infector and the infectees in space. Therefore, individual reproductive numbers of cases in the same cohort can be distinguished.

Time-varying effective reproductive numbers can be estimated by the renewal equation with a weighted ratio of infectors and infectees^11^. The *generation interval*, defined as the infection time interval between a potential infector and an infectee, is the major component of the process of determining the temporal weights of possible transmission. A major pitfall of the renewal equation method is that it automatically assumes homogeneous mixing of individuals. In effect, the temporal weight assumes that transmission probability is determined only by the temporal difference in onset of illness between the infector and infectees, regardless of possible variations due to geographical and social proximity. Human mobility and contact patterns are highly structured in the real world^16–18^ and inevitably violate the homogeneous mixing assumption^19^. Studies have demonstrated spatial heterogeneities in the transmission of dengue^20^, cholera^21^, influenza^22^, and foot-and-mouth disease^23^. Recent studies have also shown that spatial distance between cases strongly influences short-term movement^24^ and, hence, the spread of these diseases. Moreover, short-range transmission has served the essential mode of disease transmission^25^, i.e., patients typically have a higher chance of infecting others nearby. Although this spatial effect has been considered in the aforementioned studies and in the transmission tree reconstruction of some diseases^26,27^, the effect of spatial distance on the estimation of the effective reproductive number has not yet been explored. The spatial relationship between individuals can be utilized to calculate the discriminative transmission potential for each individual (Fig. 1; see the methods section for details). Neglecting the spatial variation, conversely, may smooth out heterogeneities in transmission potential, obscure high-risk spreaders, and hamper the effectiveness of control measures. In summary, the effect of spatial proximity or distance should be accounted for in the estimation of effective reproductive numbers.

Therefore, the objective of this study is to propose such method for quantifying spatially adjusted reproductive numbers that reflects spatial heterogeneity in exposure risk. It generates individual-level effective reproductive numbers (*R*^*j*^) and a summarized indicator for the whole population (*R*_*t*_) by the transmission probability estimated for all infector-infectee pairs, based on both temporal and spatial characteristics. Temporal weighting functions account for the fact that infected cases can only transmit the disease effectively within a certain time window (the generation interval). When only temporal weighting is considered, our method is equivalent to the renewal equation, assuming homogeneous mixing. The spatial weighting function, on the other hand, accounts for the decaying chance of transmission when the distance between individuals increases. The value of spatially adjusted *R*^*j*^ can provide more information regarding spatial heterogeneities in transmission potential and can better aid in the implementation of control measures. Data from dengue epidemics in southern Taiwan are used to demonstrate the ability of this method to identify early spreaders contributing to the geographic expansion of dengue transmission.

## Methods

### Study Area

**Figure 2:**
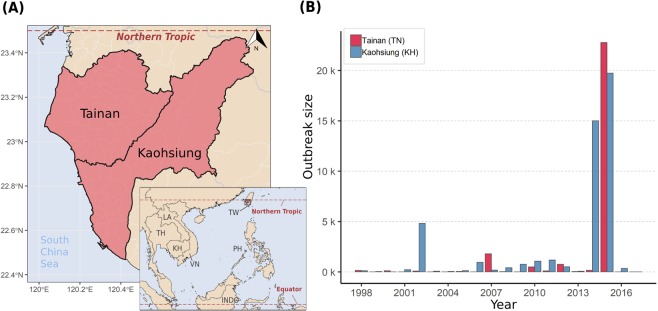
Geographic locations of Tainan (TN) city and Kaohsiung (KH) city and their history of dengue outbreaks. Panel A shows the location of the cities relative to other dengue endemic countries in Southeast Asia. Panel B shows the annual number of indigenous dengue infections recorded in TN and KH. The five most severe epidemics selected by the outbreak size were included in this study. The maps are generated by R package *ggplot2*, and *sf* (version 3.6.1, https://cran.r-project.org).

Taiwan (23.778°N, 120.930°E) is an island country at the border of tropical and subtropical climate zones. Due to its geographic proximity to dengue endemic countries in Southeast Asia (Fig. 2A), dengue outbreaks in Taiwan are triggered by imported index cases from endemic regions. Large outbreaks have occurred in the tropical monsoon regions of southern Taiwan, particularly in the cities of Tainan (TN) and Kaohsiung (KH). These two large tropical cities feature high temperatures and high humidity, high population density, and highly urbanized landscapes, all of which provide appropriate breeding habitats for *Aedes aegypti*, the main vector mosquitoes of dengue virus. Thus, as dengue outbreaks occur frequently in late summer and wane in winter, the two cities form natural settings for the repetitive observation of dengue invasion and epidemic propagation.

### Dengue data

Dengue fever is listed as a notifiable infectious disease in Taiwan. This means that physicians are mandated to report suspected cases in which the patient lives in or has a history of travel to a dengue-affected area and has corresponding symptoms, including fever, rash, eye pain, leukopenia, etc. The reported cases are then confirmed by standard laboratory tests, including real-time PCR, ELISA, and antigen detection^28^. These surveillance data are recorded and provided by Taiwan Centers for Disease Control, Ministry of Health and Welfare^29^. The dengue database starts in 1998, when the electronic reporting system was implemented, and we include all records since 1998-01-01. Individual-level information, including date of disease onset and X-Y coordinates of residence, is also provided. Each pair of residence coordinates is listed as the center of a basic statistical area, which is the smallest geographic unit for socioeconomic surveying in Taiwan. There was a total of 78,749 confirmed dengue cases (4.4% of them were imported cases) in Taiwan from 1998 to 2017, 92.5% of which occurred in Tainan and Kaohsiung cities.

Figure 2B shows the historical records of dengue epidemics in Taiwan since 1998; the five most severe outbreaks were included in this study. Among the five outbreaks, two occurred in Tainan (2007TN, 2015TN), and three occurred in Kaohsiung (2002KH, 2014KH, 2015KH). The sizes of the outbreaks ranged from 22,784 cases in 2015TN to 1,183 cases in 2011KH. Generally, dengue outbreaks start in June, reach their peak around September, and end near the end of the year or early the next January. In this study, we analyzed the largest outbreak, 2015TN, to demonstrating our method, while detailed results for the other four epidemics are presented in our supplementary results. Of the 22,784 total infections in 2015TN, only 19 cases (<0.1%) were imported from foreign countries, suggesting that indigenous transmission was well established. In contrast with Kaohsiung city, very few outbreaks occurred in Taiwan before 2015.

### Quantifying the temporal transmission dynamics

The renewal equation specifies the relationship between generations of incident cases, as shown in Eq. (1). It can be applied to estimate time-varying effective reproductive numbers due to its simplicity and generality regarding the temporal transmission dynamics^11,13,30^.

1 $${R}_{t}={\int }_{x=t}^{\infty }\frac{{\hat{b}}_{x}\,g(x-t)}{{\int }_{a=0}^{\infty }{\hat{b}}_{x-a}g(a)da}dx$$

*R*_*t*_ represents the population-level time-varying effective reproductive number at time *t*. ${\hat{b}}_{x}$ denotes the number of incident cases at time *x*, and ${\hat{b}}_{x-a}$ in the denominator denotes the number of potential infectors generated at *a* days (*a* ≥ 0) prior to time *x*. The hat notations stand for observed case numbers. *g*(*a*) stands for the distribution of the generation interval, which is used as a weighting function for representing transmission potential at time interval *a*. It can be specified by the nature of disease transmission process.

Importantly, the generation interval, also known as generation time, is the period between the infection of an infector and the infection of its infectees. It is a fundamental parameter reflecting the natural history of pathogens. Since the generation interval may vary between individuals, it can be described by a probability distribution, *g*(*a*), where *a* is a time interval. Therefore, the function can be regarded as the transmission weight between a pair of cases whose observed generation interval equals *a* (Fig. 1B). The representation of transmission weight (also referred to as transmission likelihood^15^) as *g*(*a*) is the basic component of the process of estimating effective reproductive numbers in the renewal equation and the following proposed method. The generation interval of dengue, in particular, is composed if four periods: host incubation, vector incubation, host infectious period and vector infectious period^31^. Nonetheless, showing compatibility with empirical data and mathematical convenience, gamma distributions are frequently used to model the generation interval of dengue collectively^32^. In this study, we used a gamma distribution with mean = 20 days and standard deviation = 9 days in our analysis, in accordance with a previous study^25^.

### Individual reproductive number

In order to capture spatial variations among individuals, we adopted another method developed by Wallinga and Teunis^15^ to estimate individual reproductive numbers (*R*^*j*^). We refer to this method as the non-adjusted method (i.e., non-adjusted for spatial effect). The transmission probability between pair of cases can be described mathematically as

2 $${\rm{Non}} \mbox{-} {\rm{adjusted}}\,{p}^{ij}=\frac{g({\hat{t}}^{ij})}{{\sum }_{k\ne i}g({\hat{t}}^{ik})}$$

where *p*^*ij*^ is the probability that case *j* infects case *i*. Note that we use superscripts to index time-invariant, individual (or pairwise) attributes (e.g., *R*^*j*^, *p*^*ij*^) and subscripts for time-varying attributes (e.g., *R*_*t*_). *g*(·) is the temporal weighting function, representing generation interval. Given the onset time interval ${\hat{t}}^{ij}={\hat{t}}^{i}-{\hat{t}}^{j} > 0$, $g({\hat{t}}^{ij})$ is the transmission weight of the case pair (*i*, *j*). The pairwise transmission weight is then normalized by all received transmission weights of case *i* (from all potential infectors *k* ≠ *i*) to produce consistent estimation of transmission probability. The resulting *p*^*ij*^ is interpreted as the probability of individual *i* being infected by individual *j*. Therefore, *R*^*j*^ *as* the average number of secondary cases caused by individual *j* is the sum of all *p*^*ij*^ involving *j* as the infector, as shown in Fig. 1A and Eq. (3).

3 $${\rm{Non}} \mbox{-} {\rm{adjusted}}\,{R}^{j}={\sum }_{i\ne j,{t}^{i} > {t}^{j}}{\rm{Non}} \mbox{-} {\rm{adjusted}}\,{p}^{ij}\,$$

### Spatially adjusted reproductive number

In this study, we extend a previous method with a spatial weighting function, $f({\hat{d}}^{ij})$, in order to account for the effect of spatial variation on dengue infections. We refer to this extended method as the (spatially) adjusted method. The transmission probability for case pair (*i*, *j*) becomes

4 $${\rm{Adjusted}}\,{p}^{ij}=\frac{g({\hat{t}}^{ij})f({\hat{d}}^{ij})}{{\sum }_{k\ne i}g({\hat{t}}^{ik})f({\hat{d}}^{ik})}$$

where ${\hat{d}}_{ij}$ is the distance between the pair (*i*, *j*) and *f* is a function relating transmission weight to the distance between cases. The specifications of the temporal difference ${\hat{t}}_{ij}$ and *g* remain the same as in the previous method. Likewise, the spatially adjusted individual reproductive number ${R}_{j}^{sp}$ is the sum of those ${p}_{ij}^{sp}$ involving *j* as the infector (Eq. 5).

5 $${\rm{Adjusted}}\,{R}^{j}={\sum }_{i\ne j,{t}^{i} > {t}^{j}}{\rm{Adjusted}}\,{p}^{ij}$$

The spatial weighting function is also called the transmission kernel and is a monotonically decaying function with respect to distance, reflecting neighborhood transmission^32^. Thus, unlike the generation interval, which is typically marked with a temporally lagged effect because of the latency period of infectiousness, the spatial weighting function decreases monotonically as distance increases, which means people would be easily get infections if they live near each other. It reflects people in the nearby neighborhood may share common environmental sources of dengue infection. There are several kinds of spatial kernels used in the literature, including exponential decay^25^ and power-law decay^33^. In the context of dengue transmission, we adopted an exponential decaying kernel with mean = 125 m^27^. This approach accounts for both temporal and spatial relationships when estimating individual-level reproductive numbers. Apart from the component of the generation interval inherited from the previous method, the distance-decayed spatial weighting function captures the spatial risk of dengue infection.

To calculate the population-level effective reproductive number from individual estimates, the *R*^*j*^ values can be further aggregated to form the Adjusted *R*_*t*_ given a specified time step *τ*^13^:

6 $${\rm{Adjusted}}\,{R}_{t}=\frac{{\sum }_{t-0.5\tau  < {t}^{j} < t+0.5\tau }{R}^{j}}{{\sum }_{t-0.5\tau  < {t}^{j} < t+0.5\tau }1}$$

which is simply the average of all the values of Adjusted *R*^*j*^ for which *t*^*j*^ falls in the time window [*t* − 0.5*τ*, *t* + 0.5*τ*]. As long as the weighting functions are specified, this method allows a quick estimation of population (Adjusted *R*_*t*_) and individual (Adjusted *R*^*j*^) effective reproductive numbers using observed data where onset dates are available.

### Individual transmission distance

We defined individual transmission distance *D*^*j*^ as the average of geographic distances $({\hat{d}}^{ij})$ from an infector *j* to all potential infectees *i*, weighted by their pair-wise transmission probabilities (*p*^*ij*^), as shown in Eq. (7). *D*^*j*^ could reflect the infection range transmitted by a particular infector *j*.

7 $${D}^{j}={\sum }_{i\ne j,{t}^{i} > {t}^{j}}\frac{{\hat{d}}^{ij}\,{p}^{ij}}{{\sum }_{i\ne j,{t}^{i} > {t}^{j}}{p}^{ij}}={\sum }_{i\ne j,{t}^{i} > {t}^{j}}\frac{{\hat{d}}^{ij}\,{p}^{ij}}{{R}^{j}}$$

### Quantifying the preconditions of spreaders

To explore the phenomenon of spatial expansion, two types of spreaders are of interest: *early spreaders* and *succeeding spreaders*. Early spreaders are defined as those cases that trigger a new cluster (by introducing the disease into an unaffected area). There are usually few or no cases of the disease, and the cluster tendency is low until several early spreaders emerge. Succeeding spreaders are defined as those cases that were infected by early cases nearby. Although there is a clear distinction between the two spreaders in theory, most individuals lie on a spectrum between these two extremes. We have therefore quantified this property according to the clustering tendency at the location and time immediately before the emergence of that spreader. In other words, clustering tendency is used as the measure for the precondition of a dengue case, and the median clustering tendency is used as the cutoff to classify all dengue cases. If the precondition clustering tendency is higher than the median, the case is categorized as a succeeding spreader; otherwise, it is characterized as an early spreader.

We used kernel density estimation (KDE) to quantify spatial clustering tendency in order to (1) determine the preconditions of a specific spreader and (2) compare the distributions of spatially adjusted effective reproductive numbers and case clustering patterns. The clustering tendency at the location of case *j* is defined as^34^

8 $${C}^{j}=\frac{1}{n{h}^{2}}{\sum }_{i=1}^{n}K(\frac{{\hat{d}}^{ij}}{h})$$

where *h* is called the kernel bandwidth or smoothing parameter (i.e., the parameter controlling the extent of smoothness), ${\hat{d}}^{ij}$ denotes the distance between case *i* and the locality of case *j*, and *K*(·) is a spatial smoothing function characterizing the contribution of each individual over the relative distance ${\hat{d}}^{ij}/h$.

## Results

**Figure 3:**
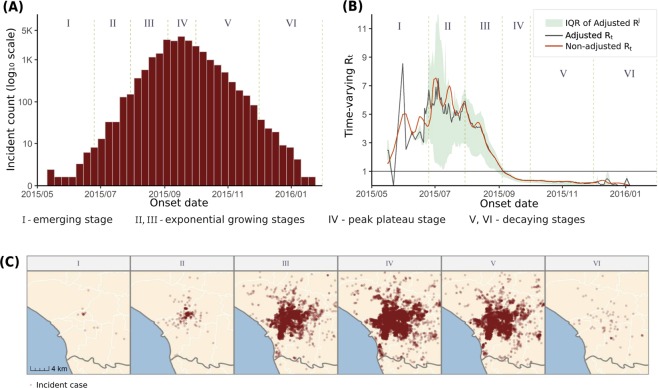
(**A**) Epidemic curve for the 2015TN outbreak, which was divided into six stages as labeled in the figure. The y-axis is log-transformed to show more clearly the incidence in in emerging stage (I) and illustrate exponential growing pattern in rapidly growing stages (II and III). (**B**) Estimated time-varying reproductive numbers through the course of the 2015TN outbreak. The curves denote the population-level *R*_*t*_ (black for spatially adjusted estimates and orange for non-adjusted estimates). The shaded area presents the interquartile range (IQR) of individual reproductive numbers (*R*^*j*^) over time, which only exists for the spatially adjusted estimates. The epidemic threshold of *R* = 1 is marked as a black, horizontal line. (**C**) Spatial distribution of incident dengue cases at the six stages. The maps are generated by R package *ggplot2*, and *sf* (version 3.6.1, https://cran.r-project.org).

The incidence of the 2015TN dengue outbreak are shown in Fig. 3A. The outbreak emerged in May with a handful of sporadic cases and finished the next January with a total of 22,784 cases. We divided the outbreak into six stages: emerging (I), growing (II and III), peak plateau (IV), and decaying (V and VI), to depict the spatiotemporal evolutions of the outbreak. Figure 3B shows the Adjusted *R*_*t*_ and Non-Adjusted *R*_*t*_ of the outbreak. These *R*_*t*_ curves are median or mean estimates of the adjusted and non-adjusted individual reproductive numbers, summarizing the temporal evolution of population-level transmission potential. Adjusted *R*_*t*_ provides further information: the shaded area represents the interquartile range (IQR) of adjusted individual reproductive numbers, reflecting the spatial individual heterogeneity of the transmission potential. In contrast, individual reproductive numbers of incident cases that shared identical dates of illness onset are constants in Non-Adjusted *R*_*t*_. This indicates that the incident cases during this period were highly geographically heterogeneous in their transmission potential. The distributions of Adjusted *R*^*j*^ in these stages are also multimodal and right-skewed, which indicates that some incident cases could have higher transmission potential (i.e. super-spreaders).

Figure 3C shows the spatial distributions of incident cases in these stages. We can identify the linkages between time-varying reproductive numbers (Fig. 3B) and spatial-temporal distributions of incident cases (Fig. 3A,C) in different stages. In the emerging stage, the irregular growth of Adjusted *R*_*t*_ reflects the initial growth of the outbreak with a sporadic distribution of incident cases and a small emerging cluster (Fig. 3C). Subsequently, in the growing stages (from stage II to III), the epidemic curve started to show exponential growth (Fig. 3A); the larger IQR of the individual reproductive numbers (Fig. 3B) reflects some incident cases with higher transmission potential that occurred and resulted in vigorous expansion of disease clustering (Fig. 3C). In the stage of peak plateau (stage IV), Adjusted *R*_*t*_ dropped under the epidemic threshold (<1), indicating that the outbreak was contained. The spatial distributions of cases also show that the clustering areas remained and stopped expanding. In sum, the time-varying reproductive numbers, Adjusted *R*_*t*_ and Non-adjusted *R*_*t*_, can reflect the timing of the outbreak containment according to the epidemic threshold. The Adjusted *R*_*t*_ can further reflect the timing of epidemic expansion when the IQR of individual reproductive numbers increases.

**Figure 4:**
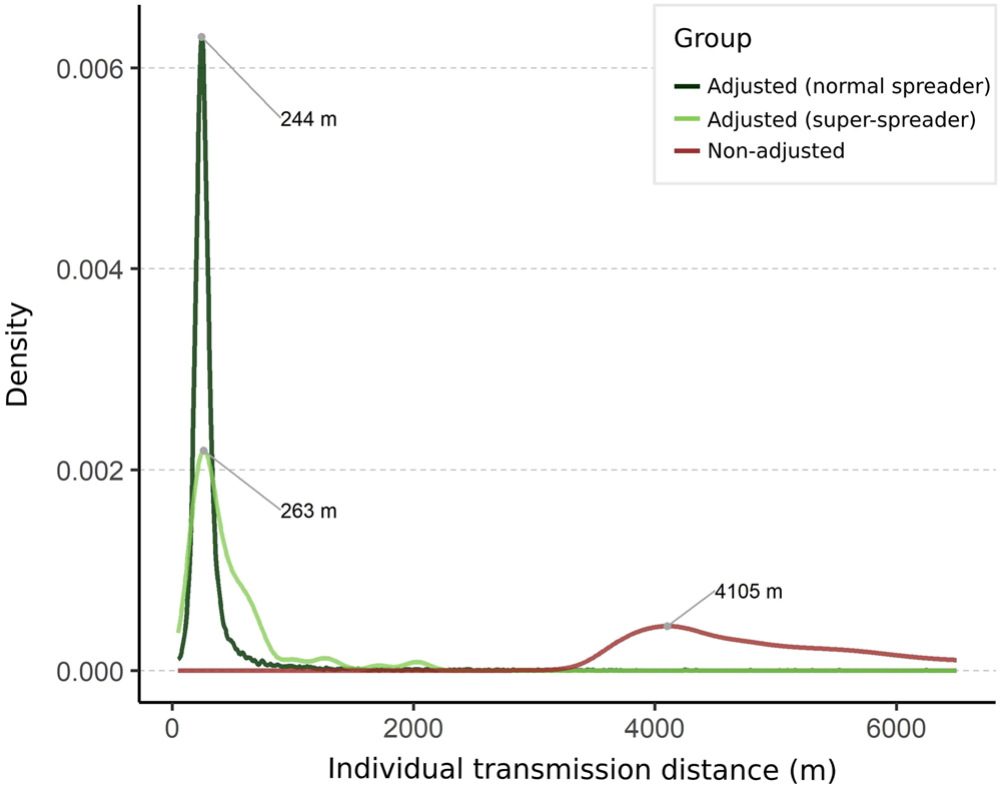
The distribution of individual transmission distance of super-spreaders (*R*^*j*^ ≥ 10) or normal spreaders (*R*^*j*^ < 10). The estimates by spatially adjusted method are shown in green (dark green for normal spreaders, and light green for super-spreaders), while the estimates by non-adjusted method are shown in dark red.

In Fig. 4, the distributions of individual transmission distance were compared between the two methods: Non-adjusted and Adjusted *R*^*j*^. Based on the neighborhood transmission setting of Adjusted *R*^*j*^, the transmission distance of an infector is around 200–300 meters and few long-range transmission links (longer than 2 kilometers). Furthermore, we used Adjusted *R*^*j*^ = 10 as a threshold to categorize super- (Adjusted *R*^*j*^ > 10) and normal- (Adjusted *R*^*j*^ < 10) spreaders. The figure also shows that long-range transmission links are from super-spreaders. The result indicates that dengue cases with high transmissibility have the ability to spread pathogens to geographically distant areas.

**Figure 5:**
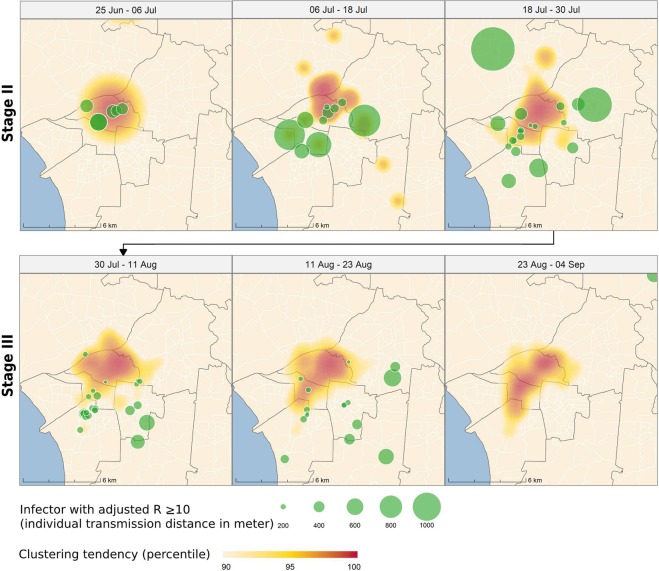
Spatial distribution of super-spreaders (*R*^*j*^ ≥ 10), compared with the main clusters of the dengue outbreak during the rapidly growing stages (II and III). The red area represents the most clustered region, the center of the ongoing outbreak, while the light yellow area represents the edge of the outbreak. The green circles represent the locations of the super-spreaders, with the radius being proportional to their transmission distances. The maps are generated by R package *ggplot2*, and *sf* (version 3.6.1, https://cran.r-project.org).

To further explore spatial relationships of super-spreaders and dengue epidemic expansion, we illustrated the locations of super-spreaders and clustering tendency of the dengue epidemic during the rapidly growing period (stages II and III), as shown in Fig. 5. Except the very beginning of the stage II (June-25–July-6), the figure shows that super-spreaders tend to distribute at or outside the edge of the main clusters from July-06 to August-23. In addition, the circle size of the super-spreaders in Fig. 5 represent their transmission range. Therefore, the locations of larger circles would reflect long-rage transmission occurred at the edge of dengue clusters. It implied that the role of super-spreaders could be the drivers of geographic expansion of the dengue epidemic.

**Figure 6:**
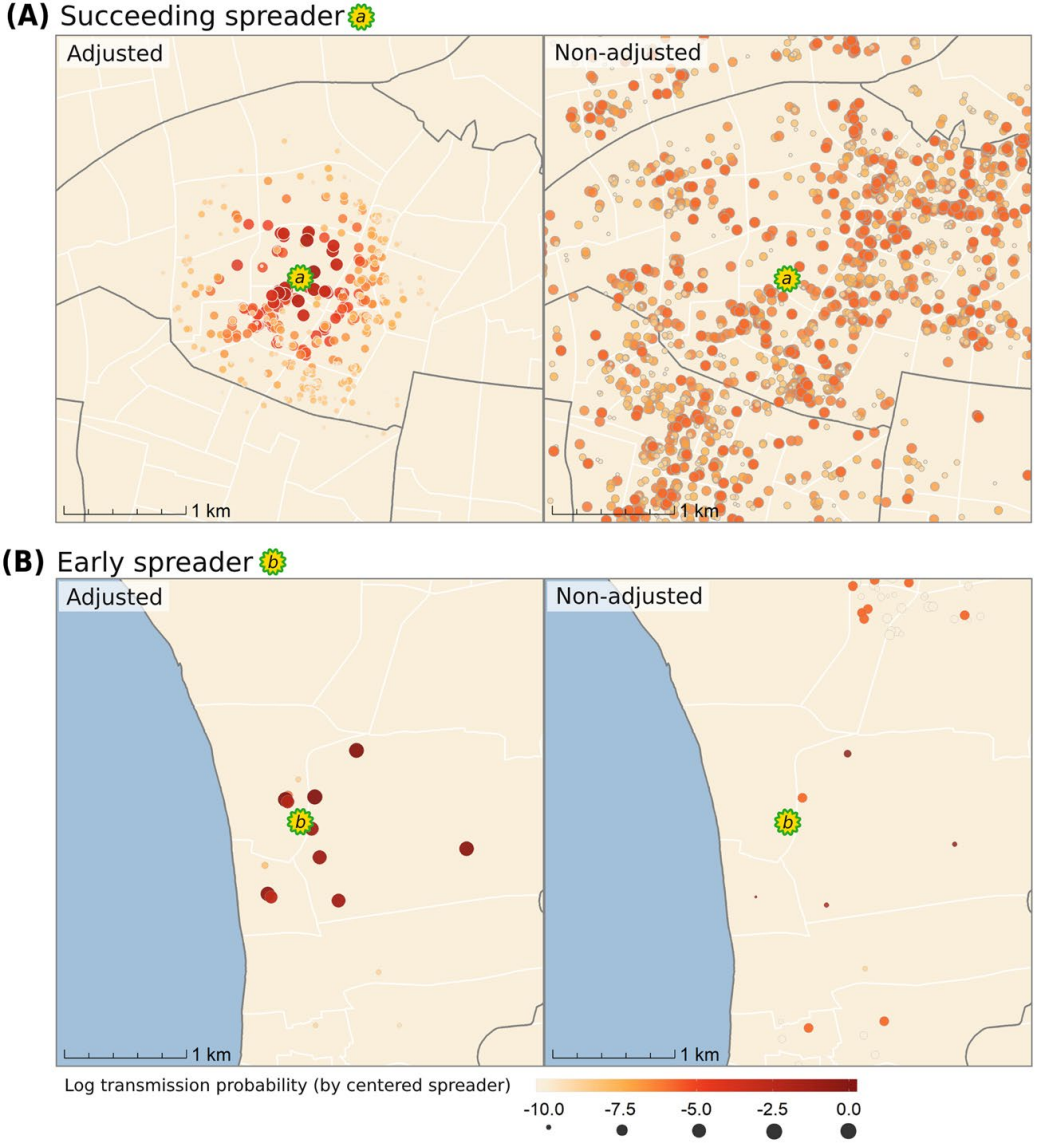
Comparisons of the transmission likelihood calculation for the two spreader types (panels A and B) between two methods. In each map, the potential infectees are shown in dots of different sizes and colors corresponding to their transmission probability by the infector (aligned at the center). (**A**) Illustration of a succeeding spreader ***a*** (Non-adjusted *R*^***a***^ = 4.15; Adjusted *R*^***a***^ = 1.85). (**B**) Illustration of an early spreader ***b*** (Non-adjusted *R*^***b***^ =4.15; Adjusted *R*^***b***^ = 5.74). The maps are generated by R package *ggplot2*, and *sf* (version 3.6.1, https://cran.r-project.org).

In order to profile the roles of different spreaders in detailed spatial transmission/expansion process, we classified the cases into two types, early spreaders and succeeding spreaders. Early spreaders are regarded as the sources of new emerging clusters, and succeeding spreaders are those that come after early spreaders. Figure 6 presents a succeeding spreader ***a*** (panel A) and an early spreader ***b*** (panel B). In each panel, we also compared different methods (spatially adjusted and non-adjusted) that estimate the transmission probability from a spreader to its potential infectees. The time of the incident cases on these maps is the 30^th^ day after the onset day of the given spreader. Infectees labeled with darker colors have a higher probability of becoming infected by spreader ***a*** or ***b***. By the non-adjusted method, the two spreaders have identical transmission probabilities and individual reproductive number because of the homogenous mixing assumption (Non-adjusted *R*^***a***^ = Non-adjusted *R*^***b***^ = 4.15). However, they should play different roles in the spatial expansion of the outbreak. Spreader ***a*** (the succeeding spreader) occurred in an ongoing cluster; thus, this case is unlikely to be the primary source that triggered this local outbreak. Spreader ***b*** (the early spreader), on the contrary, initiated a new cluster where no case had occurred before, and the following cases that emerged were centered on spreader ***b***. Thus, a subsequent local outbreak can be logically attributed to spreader ***b*** as the primary ancestor. The adjusted method (left-side maps in Fig. 6), which takes into account the spatial-temporal relationships of incident cases, differentiates the individual reproductive numbers of early and succeeding spreaders (Adjusted *R*^***a***^ = 1.84, Adjusted *R*^***b***^ = 5.74). It also yields more reasonable spatial transmission potential by upweighting the potential infectees proximity to spreader ***a*** in Fig. 6A.

**Figure 7:**
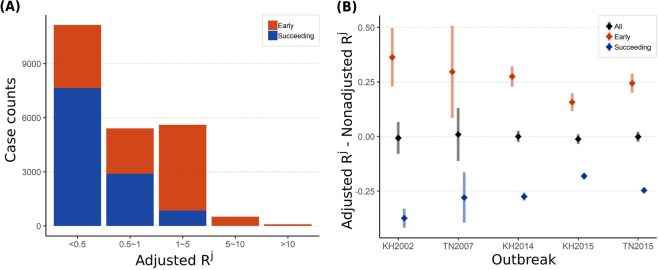
(**A**) The individual-level spatially adjusted reproductive numbers (Adjusted *R*^*j*^) of early and succeeding spreaders. (**B**) Paired difference in the estimates of the individual reproductive number (Adjusted *R*^*j*^–Non-Adjusted *R*^*j*^) by different spreader types and major outbreaks.

To clarify the distinct roles of early and succeeding spreaders in the outbreak expansion process, we compared the distributions of Adjusted *R*^*j*^ between different types of spreaders in Fig. 7A. We found that all the super spreaders (Adjusted *R*^*j*^ > 10) are early spreaders and most of succeeding spreaders have low Adjusted *R*^*j*^. To examine the generalization of this pattern, we estimated Adjusted *R*^*j*^ of dengue cases in top five largest dengue epidemics since 1990 in Taiwan, including KH2002, TN2007, KH2014, KH2015, TN2015, as shown in Fig. 7B. Non-adjusted *R*^*j*^ were used as the baseline to control for the fluctuations across different stages of the outbreak. The figure showed consistent patterns among these large-scale dengue outbreaks, which means early spreaders with high transmissibility can be generally highlighted by the spatially adjusted method.

## Discussion

The effective time-varying reproductive number is a commonly used indicator for measuring disease transmissibility. However, the index conventionally does not capture spatial dynamics of disease transmission. We proposed a new method of calculating the spatially adjusted effective reproductive number by incorporating a spatial-weighting function that captures the nature of heterogeneous mixing. Unlike the averaged time-varying reproductive number, this method estimates different individual-level reproductive numbers (*R*^*j*^) for given onset times and locations of dengue infections. Thus, it can reflect spatial heterogeneity in transmission potential among individuals and identify the possible super-spreaders^35^ with high *R*^*j*^ during the rapidly growing period. Our results also reveal that dengue cases with high transmission potential and long-transmission distance are usually located at the edges of the epidemic foci, which means they could be the drivers of further outbreak expansion. Therefore, our proposed method depicts a more detailed spatial-temporal dengue transmission process and identifies the significant role of the edges apart from the epidemic foci, which could be the weak spots in disease control and prevention. Our spatially adjusted method also could further apply to assess individual-level transmission potential of other acute contagious diseases, such as influenza and Zika virus infection, with observed generation interval and the context of neighborhood transmission.

The effective reproductive number of dengue as estimated in past studies reflected an averaged overall epidemic trend, which did not take into account the spatial heterogeneity of transmissibility. Hsieh^36^ estimated the effective reproductive number at the initial stage of the 2015TN outbreak to be 6.84, which is similar to our averaged estimates (Fig. 3B). Hsieh’s study also showed that Kaohsiung consistently possessed lower effective reproductive numbers (from 1.29 to 2.87), implying that Tainan city may have epidemiological characteristics, such as a lack of herd immunity, that make it more prone to dengue transmission than Kaohsiung. Internationally, Guzzetta^25^ reported that the time-varying reproductive number of non-endemic urban cities in Brazil is much smaller than the estimates from Tainan and Kaohsiung cities (maximum *R*_*t*_ is approximately 2.2). Codeco^31^ and Pinho^37^ also estimated time-varying reproductive numbers in Salvador and Brazil, with a maximum *R*_*t*_ of approximately 4.5. Comparing the values of *R*_*t*_ among different cities may be subject to possible confounding factors, such as weather conditions, host immunity and circulating viral strands.

Some studies considered location information to determine reproductive numbers for quantifying transmissibility of foot-and-mouth disease (FMD)^38,39^. These models considered spatial distribution of farms, however, it was difficult to capture FMD disease transmission process among animals. In other words, the time-varying reproductive numbers in these models are difficult to reflect nature course of FMD transmission in terms of implementing general interval and renewal equation. Furthermore, farm-level models for FMD outbreaks can be categorized the farms into infected and susceptible ones to determine relative transmissibility of each farm at a specific time. It may be difficult to estimate the amount of susceptible persons living around the infected cases for human infectious diseases, such as influenza and dengue fever. Other studies have also explored spatial heterogeneity by performing stratified analysis of *R*_*t*_ with respect to different administrative regions and with respect to region-to-region transmission^40,41^. These studies used a spatial weighting function to emphasize the interregional transmission process but still assumed a homogeneous-mixing model within each region. Thus, these studies did not address the spatial heterogeneous mixing issue when estimating reproductive numbers. Our study considered individual-level spatial heterogeneity and used the spatially adjusted reproductive number to measure the transmission potential of each individual.

Like the basic reproductive number, *R*^*j*^ can be regarded as a function of duration of infectiousness, incubation period, transmission probability, vector mosquito density, and host-vector contact rate. The adjusted *R*^*j*^ in this study could reflect vector mosquito density and host-vector contact rate, which are also highly heterogeneous in space^42^. Meanwhile, the dispersal of vector mosquitoes is largely confined to neighboring areas (average radius of 28–199 meters^43^), providing an effective infectious zone of an infector. An exponential spatial weighting function herein represents this infectious zone. Guzzetta^25^ estimated the mean transmission distance of dengue in a metropolis area to be approximately 127 m and further indicated that an exponential distribution described the data better than a radiation model which is a more dispersed distribution effectively describes human mobility^44^. Kissler, *et al*.^45^ also reported the aptness of the exponential distribution when an outbreak is typically dominated by short-range transmission. Therefore, the exponential spatial weighting function in this study is an appropriate substitute for the effective infectious zone for measuring dengue transmission. The exponential distance-decayed function also avoids overestimating the transmission probabilities of infector-infectee pairs with long geographic distances, especially for large-scale dengue epidemics. In sum, areas with high *R*^*j*^ are potential risk areas for high dengue transmission; knowledge of such areas is important for spatial targeting during dengue epidemics.

Spatial epidemiological studies focused for many years on developing methods for identifying significant disease clustering in time and space, such as space-time scan statistics^46^ or point pattern analysis^47^. Hotspot areas identified are usually regarded as significant risk areas and as high-priority sites for intervention strategies aimed at mitigating an epidemic^48,49^. The significance of the study is to introduce the perspective of individual-level transmission potential to disease risk mapping. We found that locations of individuals with high transmission potential are usually located at the edges of growing disease clusters, which can easily be neglected when intervention resources focus on epidemic clusters. A previous study found that urban villages that were originally at the edge of the city but are now enclosed by urbanized lands act as transfer stations for dengue outbreaks^50^. In this study, we further provide a better understanding of outbreak expansion by categorizing different types of spreaders. Early spreaders with high transmission potential may initiate new source of infection at the edges of the main cluster, resulting in geographic expansion at the exponential growing stages of the outbreak. Therefore, the edge of the outbreak should be a priority of spatial targeting to contain the outbreak regarding both range and magnitude. Succeeding spreaders are indeed still important in tallying morbidity and fatality. However, their high-density clustering patterns make them prone to the depletion of local susceptible populations and degenerating transmission potential. In summary, the center and edges of epidemic clusters play different roles in developing epidemic progression in terms of different types of spreaders (succeeding vs. early) and different patterns of epidemic growth (intensifying vs. expanding). These findings provide important insights for implementing different interventions in the center and on the edges of epidemic clusters.

There are several limitations to this study. First, the method of estimating time-varying effective reproductive numbers is a retrospective procedure that uses observed infectee generation to estimate the *R*^*j*^ of the infector generation. In other words, it cannot be used for predicting future epidemic progression in real time. Nonetheless, the method is helpful for understanding the course of an epidemic and studying the possible mechanisms of geographical expansion. Second, this study considers only geographical distance as a factor in transmission potential. Other factors influence the spatial spreading of dengue. Among these, host heterogeneity (including variations in density^51^, mobility^52^, and susceptibility^53^) strongly modulates the transmission dynamic^42^ and should be considered in further studies. Finally, the spatial weighting function reflects the assumption of distance-decayed properties (neighborhood transmission). However, the assumption may not reflect long-distance transmission^54^ and complex urban transport and mobility^52^. The question of how to develop more detailed spatial weighting schemes that capture realistic mobility patterns warrants further investigation.

## Supplementary information

Supplementary Information

## Data Availability

The dengue surveillance dataset used in the current study are publicly available in Taiwan CDC Open Data Portal, https://data.cdc.gov.tw. The data analysis tutorial is included in Supplementary Information files.
