# Supplementary material for: Spatially Adjusted Time-varying Reproductive Numbers: Understanding the Geographical Expansion of Urban Dengue Outbreaks
Source: Sci Rep. 2019 Dec 16;9:19172. doi: 10.1038/s41598-019-55574-0 (PMC6914775; doi:10.1038/s41598-019-55574-0)

**Supplemental Results**

**Spatially Adjusted Time-varying Reproductive Numbers: Understanding the Geographical Expansion of Urban Dengue Outbreaks**

Ta-Chou Ng, Tzai-Hung Wen

**[Figure S1]** Kaohsiung city (KH2002) outbreak. (A) Incident cases over the course of KH2002 outbreak, which is divided into eight periods. (B) Time-varying reproductive number estimated by non-adjusted (orange) and spatially adjusted (black) methods. The shaded area represents individual- heterogeneity in adjusted reproductive number. (C) Spatiotemporal distribution of infected cases through the eight periods.


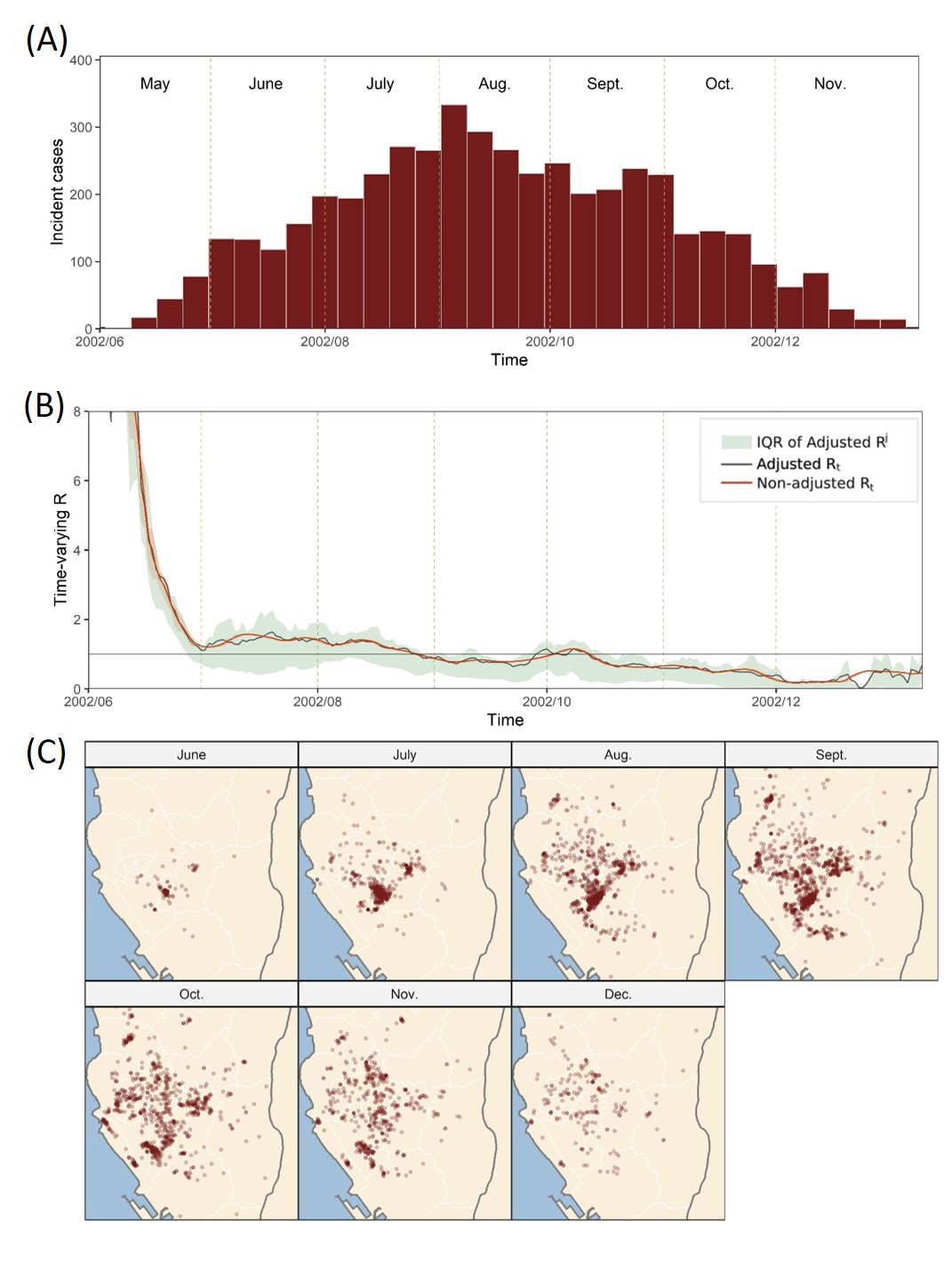


**[Figure S2]** Kaohsiung city (KH2014) outbreak. (A) Incident cases over the course of KH2014 outbreak, which is divided into eight periods. (B) Time-varying reproductive number estimated by non-adjusted (orange) and spatially adjusted (black) methods. The shaded area represents individual- heterogeneity in adjusted reproductive number. (C) Spatiotemporal distribution of infected cases through the eight periods.


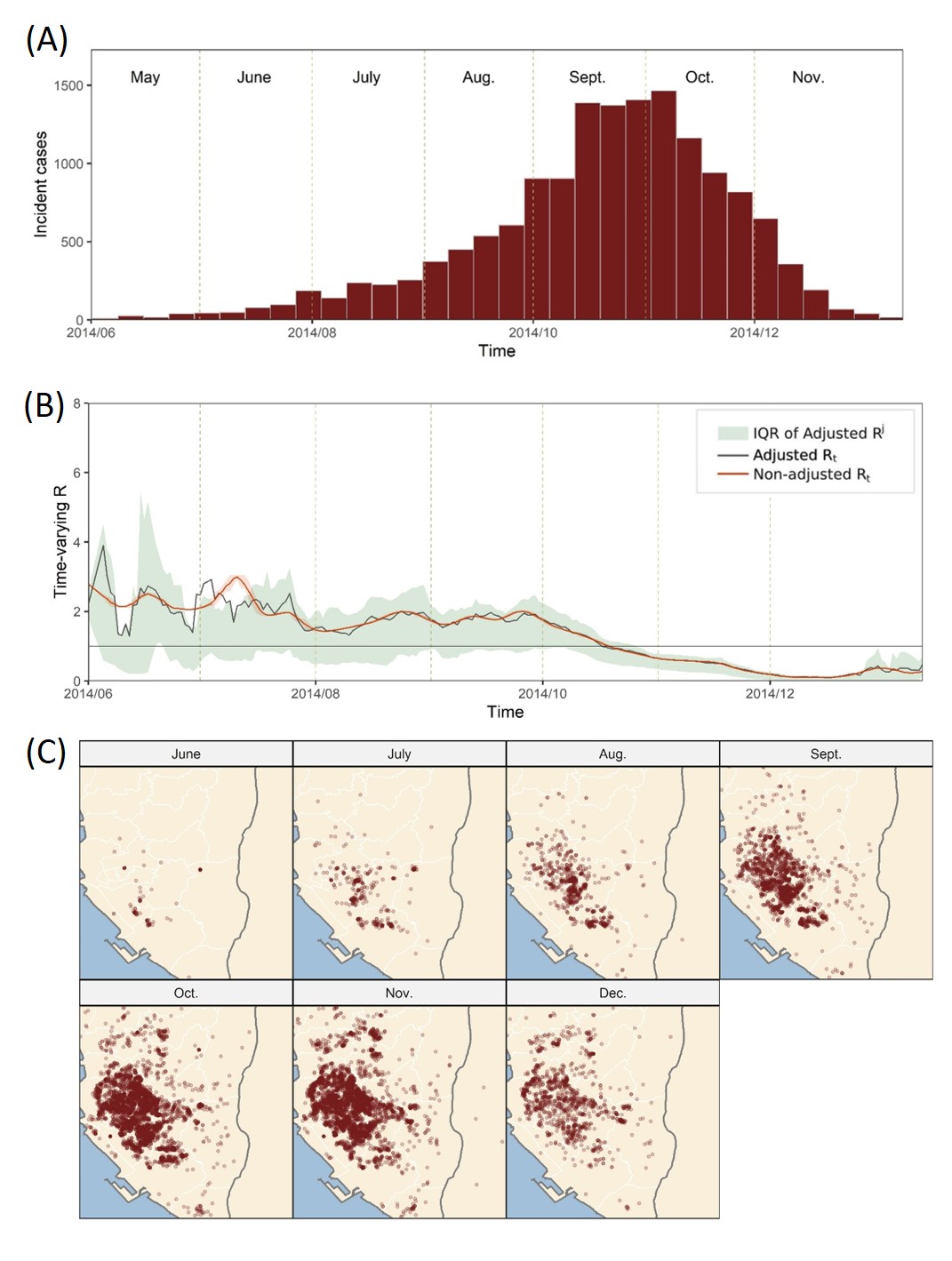


**[Figure S3]** Kaohsiung city (KH2015) outbreak. (A) Incident cases over the course of KH2015 outbreak, which is divided into eight periods. (B) Time-varying reproductive number estimated by non-adjusted (orange) and spatially adjusted (black) methods. The shaded area represents individual- heterogeneity in adjusted reproductive number. (C) Spatiotemporal distribution of infected cases through the eight periods.


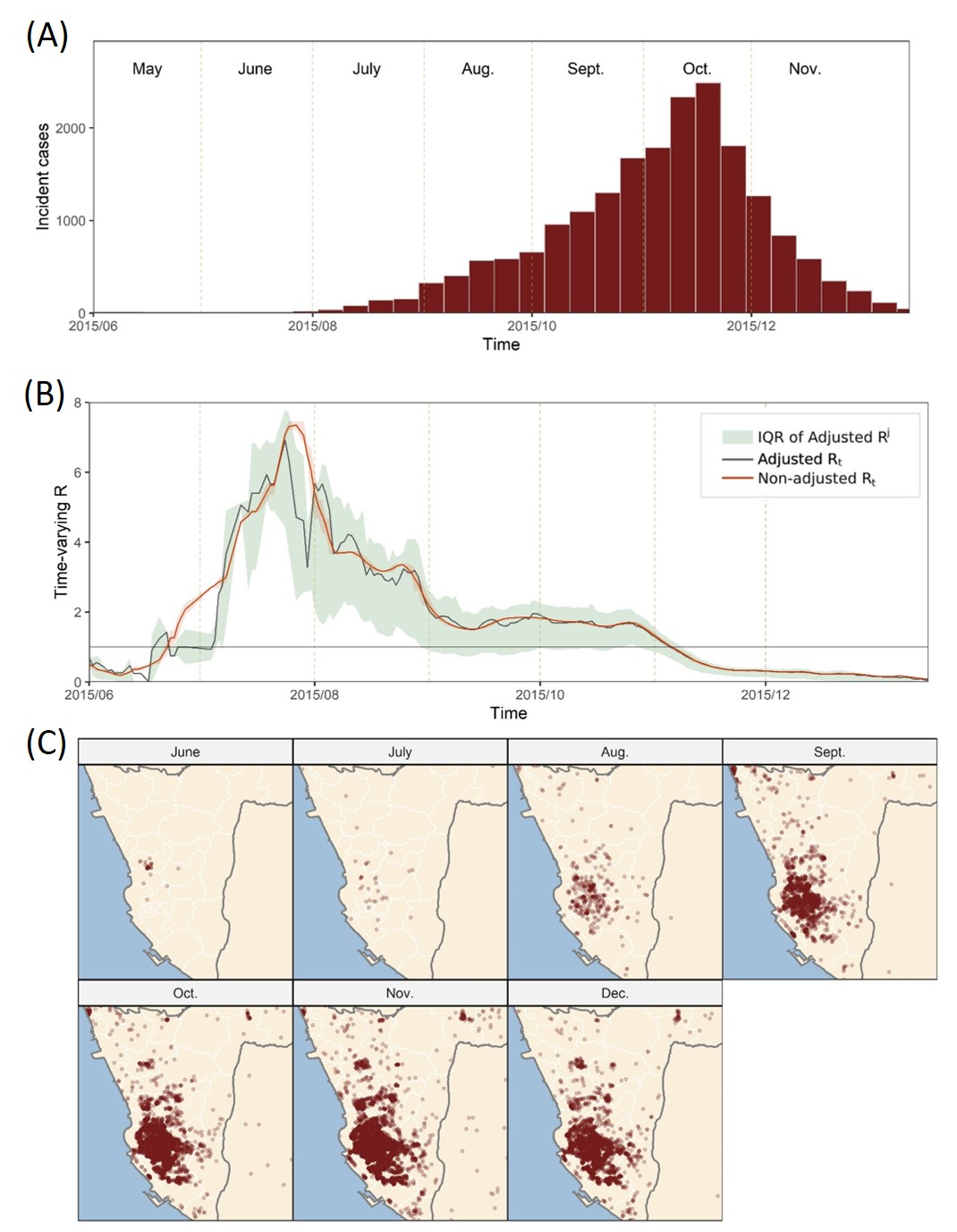


**[Figure S4]** Tainan city (KH2007) outbreak. (A) Incident cases over the course of TN2007 outbreak, which is divided into eight periods. (B) Time-varying reproductive number estimated by non-adjusted (orange) and spatially adjusted (black) methods. The shaded area represents individual- heterogeneity in adjusted reproductive number. (C) Spatiotemporal distribution of infected cases through the eight periods.


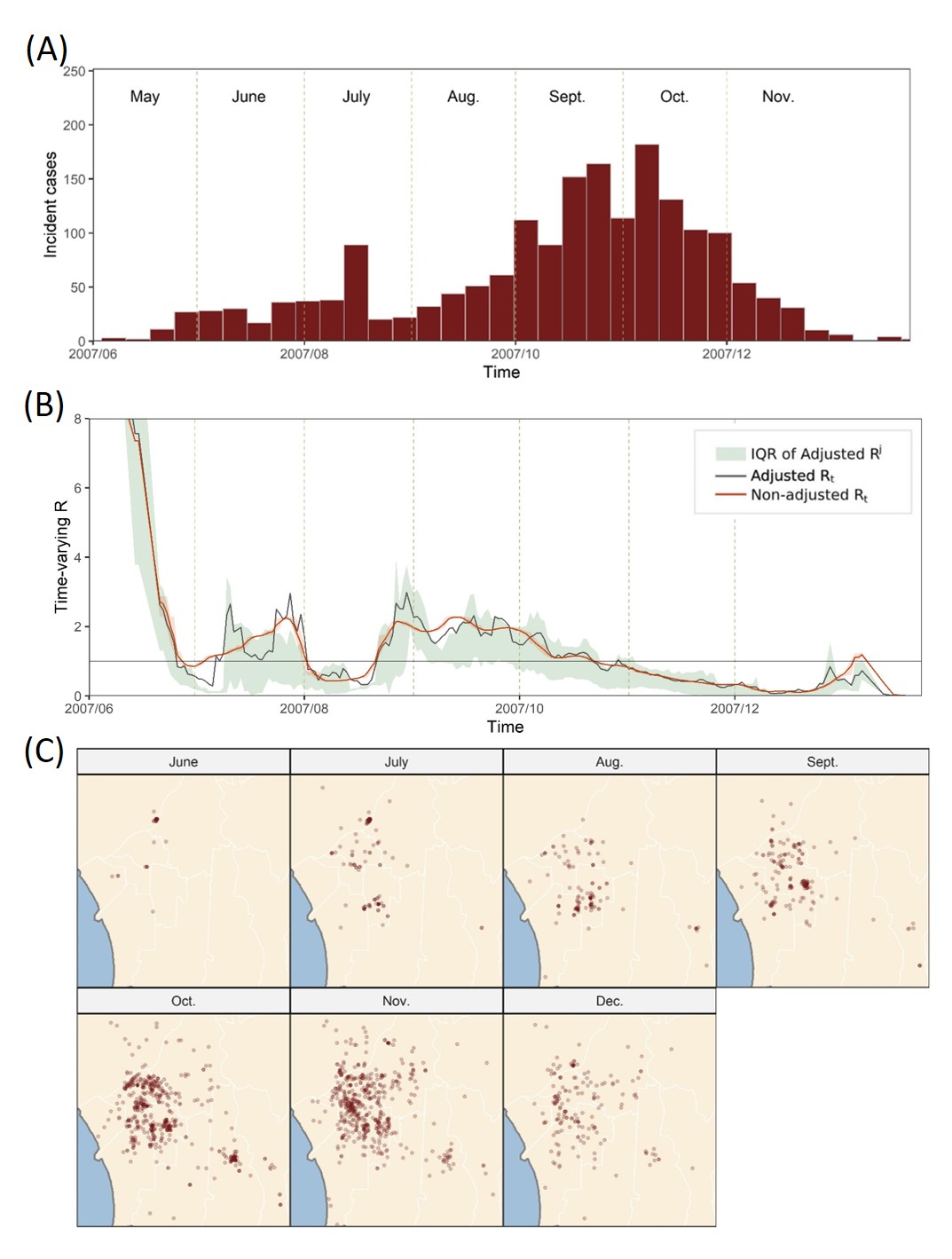

Supplement: Supplementary file 1 — Supplementary Information [file 41598_2019_55574_MOESM1_ESM.docx]
